# A System of Gynæcology

**Published:** 1908-09

**Authors:** 


### BOOKS ON GYNÆCOLOGY.

**A System of Gynæcology.** By many writers. Edited by Thomas Clifford Allbutt, M.A., M.D., W. S. Playfair, M.D., and Thomas Watts Eden, M.D. Pp. xx., 949. London: Macmillan and Co., Limited. 1906.

A review of such a work as this within the compass of a short paragraph is a practical impossibility, as it is impossible to give a really critical survey of each of the articles by recognised authorities which it contains. We can only point out the articles which will best repay perusal, and indicate some of the points in them which, perhaps, demand criticism or special notice.

From the historical point of view, one of the most interesting is that on the development of modern gynæcology by Dr. Montagu

Handfield-Jones, in which the gradual adjustment of pathological facts and clinical observations has resulted in progressive correction of errors, and consequent alterations in teaching which, from the point of view of the student, is extremely important. There can be no doubt that progress of this kind, based as it is on pathological facts, is of the very first importance.

Another article which should be read by every gynæcologist is that on Chorionepithelioma Malignum by T. W. Eden and J. H. Teacher. This may be looked upon as practically embodying the latest researches in connection with the disease.

It is impossible to give in detail all the information, much of which is either new or a collection of various older observations, which is here brought into line and compared. One point worth noticing is brought out by Teacher's series of cases, that this disease follows abortion or hydatidiform mole in approximately two-thirds of the cases collected by him. This indicates that the character of the pregnancy is by no means an unimportant factor in the production of the disease.

Another interesting point which Teacher has brought out is the fact that the metastases, which clinical observations prove to be present, frequently disappear after the removal of the primary growth by radical operation, and even occasionally after its partial or incomplete removal by curetting. This indicates extraordinary variations in the malignancy of the growth under various circumstances, and would strike us as being possibly capable of affording some kind of clue to the variations in malignancy which occur in the other better known forms of malignant disease. The article, however, should be read in its entirety to be fully appreciated.

The articles on "Disease of the Fallopian Tubes" by Mr. Alban Doran, "Pelvic Inflammation" by the late Dr. Cullingworth, "Pelvic Hæmatocele" by Dr. G. F. Blacker, and "Extra-Uterine Gestation" by Mr. Bland-Sutton will also repay careful perusal.

With reference to Dr. Blacker's article on "Hæmatocele," the following interesting observations are recorded, which we think all modern experience supports. For example, Fritsch has never seen a case of hæmatocele in which the presence of tubal pregnancy could be excluded with absolute certainty. Schauta has observed the condition only as the result of tubal abortion. Fehling states that its cause is tubal pregnancy in 90 to 95 per cent. of cases.

Cullingworth in 25 cases in which the source of bleeding could be verified found 24 to be due to tubal pregnancy, the 25th being due to rupture of a cyst of the broad ligament associated curiously enough with tubal pregnancy also. These figures give the almost unvaried experience of those who have by operation or autopsy verified the sources of hemorrhage in pelvic hæmatocele.

Blacker remarks that figures which tend to prove the opposite (*e.g.* those of Oberer, Weiss and Spengler) must be accepted with caution, as in the majority diagnosis was not confirmed by operation or autopsy. While hæmatoceles due to tubal pregnancy may be regarded as a rule, this, like other rules, is not one which is without exception. Exceptions are, however, far more infrequent than has been supposed. As regards treatment in this condition, Blacker evidently leans, and we think rightly so, to the more active modern treatment by operative methods.

The article by the late Dr. Cullingworth will tend, we think, to clear up and place on a more satisfactory basis the teaching as regards pelvic inflammation. He shows how, in spite of the observations of Aran and Bernutz, ideas entirely erroneous as to the relative frequency of inflammation of the cellular tissue and peritoneum of the pelvis have been promulgated for many years. His records of the relative frequency of these two affections can only be the result of either autopsy or operative treatment, and the tendency of all work in this direction has been to show the relative frequency of peritoneal as compared with cellulitic inflammation of the pelvis.

The article on "Antisepsis and Asepsis," by Dr. T. W. Eden, is also a valuable contribution to surgical knowledge. We, however, take exception to one statement of the author's, that is, as to the necessity or the reverse of high pressure sterilisation in the case of operations on private patients. While admitting that this is a necessity in hospital practice, he does not consider it to be of such prime importance in the operative treatment of patients in private practice of better social standing. Although there is no doubt that in the case of the latter success will result from methods the standard of which is not of the highest, we are quite unable to see why, if the methods advocated for hospital patients *do* give an increased margin of safety, these should not be made use of in the case of private patients, since in our experience there is no fundamental difficulty in carrying out precautions in their case deemed necessary in the case of patients of the former class.

The article by Professor Herbert Spencer on "Abdominal Hysterectomy" should also be read with care by those who are in the habit of performing this operation. We think that it would have been improved by a more detailed account of the methods of repair found most useful in cases in which the ureter has been injured. This accident, although not a frequent one, is probably one which few operators escape altogether; and in view of the importance of technical detail in dealing with its results, we think the account of this could not be too full or explicit.

Space will not allow of a more detailed account of the many interesting and valuable papers included in this work, which may be looked upon as a compendium of the latest methods in gynæcological practice. We can only say that from the point of view of the specialist, the practitioner or the senior student, especially the candidate for the higher examinations, this work is invaluable.

